# E2A Predicts Prognosis of Colorectal Cancer Patients and Regulates Cancer Cell Growth by Targeting miR-320a

**DOI:** 10.1371/journal.pone.0085201

**Published:** 2014-01-13

**Authors:** Ao Huang, Hongchao Zhao, Yingjun Quan, Runsen Jin, Bo Feng, Minhua Zheng

**Affiliations:** 1 Department of Surgery, Ruijin Hospital, Shanghai Jiao Tong University School of Medicine, Shanghai, People's Republic of China; 2 Shanghai Institute of Digestive Surgery, Shanghai, China; 3 Shanghai Minimally Invasive Surgery Center, Shanghai, China; University of Alabama at Birmingham, United States of America

## Abstract

**Background and objective:**

Transcriptional factor E2A is crucial for the normal development and differentiation of B and T lymphocytes. Dysregulation of E2A leads to leukemia and tumorigenesis of some solid tumors. The expression and clinical significance of E2A as well as its role in colorectal cancer (CRC) are still unknown. This study aims to assess E2A expression in CRC tissues, evaluate its prognosis value, and investigate its role in colon cancer cell growth.

**Methods:**

E2A expression in CRC tissues and normal mucosa was detected by immunohistochemical staining; Kaplan-Meier survival curve and Cox regression model were used to evaluate the prognostic value of E2A. Lentivirus was used to construct E2A stably knocked-down cells. MTT assay was employed to detect cell proliferation change; cell cycle was analyzed by flow cytometry; and chromatin immunoprecipitation (ChIP) assay was used to validate the predicted binding target of E2A.

**Results:**

Expression of E2A was lower in CRC tissues than normal mucosa; low E2A expression correlated with advanced TNM stage and larger tumor size, and predicted poor prognosis of CRC patients. E2A knockdown resulted in increased cell proliferation rate and cell cycle acceleration. ChIP assay showed miR-320a was a direct target of E2A and upregulation of miR-320a in E2A downregulated cells could reverse cell proliferation and cell cycle changes caused by E2A deficiency.

**Conclusions:**

E2A is an independent prognostic factor for CRC patients and targets miR-320a to regulate cell proliferation of colon cancer cells.

## Introduction

The mammalian E2A gene is located at chromosome 19 and is non-tissue-specifically and ubiquitously expressed in a wide range of cell types. Through alternative splicing, the E2A gene encodes two isoforms, E12 and E47 (collectively referred as E2A proteins), which both have the basic-Helix-Loop-Helix (bHLH) domain and could regulate gene transcription by binding to the E-box DNA sequences, CAGGTG. Though widely expressed, E2A is not essential in some organogeneses like skeletal or cardiac myogenesis, erythropoiesis, chondrogenesis, and neurogenesis [1] but plays an important role in the development and differentiation of B [2], [3] and T lymphocytes [4]. E2A deficient mice showed an arrest at the pro-B cell stage during B cell development [3] and transgenic expression of either E12 or E47 could partially rescue the B lymphopoiesis initiation; similarly, E2A deficiency led to a block at the earliest stage of T cell development [4] and disturbed thymocyte positive selection [5]. Moreover, E2A has been found to be involved in some cellular activities including cell differentiation [6], proliferation [7], apoptosis [8], cell cycle [9] and epithelial-mesenchymal transition (EMT) [10].

Besides its crucial role in normal B and T cell development, E2A also participates in tumorigenesis. Studies had reported the well-established role of E2A in leukemogenesis: two fusion proteins, E2A-HLF [11] and E2A-PBX1 [12], both containing the transactivation domain of E2A and the DNA-binding domain of HLF or PBX1, could lead to pro-B cell acute lymphoblastic leukemia (ALL) in adolescents and pre-B cell ALL in children respectively [13]. Moreover, E2A has been found to be involved in the oncogenesis of solid tumors, either as oncogene or as tumor-suppressor gene. Presence of E2A-PBX1 fusion protein in lung cancer was recently reported and it correlated with overall survival of patients with lung adenocarcinoma in situ [14]. Increased expression of E2A was detected in breast cancer [15], [16] and prostate cancer [17], of which it was found to promote oncogenesis. However, mice with null mutation of E2A were susceptible to spontaneously developed thymic lymphomas [18] and loss of E2A in primary effusion lymphoma led to apoptosis resistance [19], suggesting the alternative role of E2A as a tumor-suppressor gene. Furthermore, E2A expression has been proposed to be of diagnostic value in certain subtype of gastric MALT (mucosal-associated lymphoid tissue) lymphoma [20]. Taken together, E2A may act as a tumor-suppressor gene or as an oncogene in different cancers.

The expression of E2A in colorectal cancer (CRC) and its prognostic value have not been discussed before and it’s still unknown whether E2A promotes or represses the development of CRC. In this study, to our knowledge, for the first time we investigated the clinical significance of E2A in CRC and found its usefulness as a prognostic marker; furthermore, we found E2A suppressed tumor growth by targeting miR-320a in colon cancer cells, demonstrating its tumor-suppressive role in CRC.

## Methods

### Patients and clinical specimens

A total of 98 colorectal cancer patients were included; the patients were treated with surgery between June 2007 and January 2008 at Shanghai Minimally Invasive Surgery Center. Patients were excluded if they: had received neoadjuvant chemoradiotherapy; had unresectable colorectal cancers; had tumors of other organs; were unlikely to be interviewed during the follow-up. Demographic and clinicopathological data were extracted by chart review. Patients were interviewed by telephone at three months, six months, and then annually after surgery. Tumor samples were cut immediately after surgical specimens' removal during the operations and then fixed with formalin and preserved at 4°C for two weeks before next treatment; normal tissues were defined as mucosa located at least 5cm away from the tumor margin and cut, fixed, preserved and treated as above. This study was conducted with the written informed consents of all enrolled patients before surgery and under the protocol approved by the Ethical Committee of Ruijin Hospital Shanghai Jiao Tong University School of Medicine.

### Immunohistochemistry and scoring

Immunohistochemistry staining was performed as protocol previously described [21]. Briefly, after fixation with formalin, tissues were embedded with paraffin, cut, and mounted on slides. Then, slides were washed with xylene to deparaffinize, with graded ethanol to rehydrate, incubated with citrate buffer to retrieve antigen, and blocked with 3% H_2_O_2_ to inactivate endogenous peroxidase. Slides were incubated with E2A primary antibody (Santa Cruz Biotechnology, Texas, USA; 1∶200 dilution) at 4°C overnight, followed by incubation with horseradish peroxidase (HRP)-conjugated goat anti-rabbit secondary antibody (Santa Cruz) for 30 minutes at room temperature. Complex visualization was done with a 2-Solution DAB Kit (Invitrogen). Negative controls were obtained by replacing the E2A primary antibody with preimmune rabbit serum.

Slides were examined by two researchers (Huang and Quan) independently. Scoring criteria used were as previously described with minor modifications. Staining intensity was scored as 0 (no staining), 1 (weak staining), 2 (moderate staining), and 3 (strong staining); positive cells on each section were scored as 0 (less than 10%), 1 (10%-25%), 2 (26%–50%), and 3 (>50%). The final score was a product of scores of intensity and positive cell of each slide. Slides with score 0–3 were defined as low expression and 4–9 as high expression.

### Cell culture

Colon cancer cell lines LOVO, HCT116, Caco-2, HT29, SW480, and SW1116 were obtained from American Type Culture Collection (ATCC) and subcultured and preserved by Shanghai Institute of Digestive Surgery; normal human colon mucosal epithelium cell NCM460 was kindly donated by Jingqing Zeng (Ruijin Hospital, Shanghai Jiao Tong University School of Medicine; the donator bought NCM460 cell line from INCELL Corporation, LLC, San Antonio, TX, USA). LOVO was cultured in F-12K Nutrient Mixture Medium (Corning Cellgro®, MA, USA), HCT116 and HT29 in McCoy's 5A (Corning Cellgro®), SW480 and SW1116 in Leibovitz' L-15 (Corning Cellgro®), and NCM460 in DMEM (Corning Cellgro®). All culture medium above was supplemented with 10% fetal bovine serum (FBS) (Invitrogen, Carlsbad, CA, USA). Caco-2 was cultured in Minimum Essential Medium (Corning Cellgro®) with 20% FBS. Cells were placed in the incubator (Heracelles, Germany) at 37°C, in a humidified atmosphere with 5% CO_2_.

### Protein extraction and Western blot

Cells were harvested at a confluence of 80% with RIPA (Solarbio, Beijing, China) and the protein concentration was determined with BCA Protein Assay Kit (Thermo Scientific, USA) by measuring OD_562_ using microplate reader (Epoch, BioTek, Winooski, USA). Western blot was performed according to previously reported protocol [22] and primary antibodies used were E2A (1∶500, Santa Cruz Biotechnology, Texas, USA). GAPDH (Kangchen, Shanghai, China) was used as loading control.

### RNA and microRNA isolation, qRT-PCR

Total cell RNAs were extracted using Trizol reagent (Invitrogen) and total cell microRNAs (miRNAs) were extracted with mirVana™ miRNA Isolation Kit (Applied Biosystems, Foster City, CA, USA). RNA reverse transcription was performed by using PrimeScript RT Master Mix Perfect Real Time (TaKaRa, Shiga, Japan). The mRNA level of E2A (forward: 5′-CCACT TCACT GAGTC GCACAG-3′; reverse: 5′-GTCTC TCCCG AAGGA GGCATA-3′) was evaluated by qRT-PCR, using SYBR Green PCR Master Mix (Applied Biosystems); the RNA level of GAPDH (forward: 5′-GGAGC GAGAT CCCTC CAAAAT-3′; reverse: 5′-GGCTG TTGTC ATACT TCTCA TGG-3′) was used for normalization. The expression of miR-320a was evaluated by qRT-PCR using Taqman microRNA RT Kit (Applied Biosystems, Foster City, CA, USA), TaqMan MicroRNA Assays (Applied Biosystems), and Taqman universal PCR master mix (Applied Biosystems, Foster City, CA, USA), in accordance with the manufacturers' instructions; the U6 miRNA level was used for normalization.

### Lentiviral transfection for stable expression clones

E2A/shRNA-eGFP-lentivirus particles and E2A/sh-negative control (shNC)-eGFP-lentivirus particles, namely E2A/shRNA-LV and E2A/shNC-LV, were purchased from Novobio (Shanghai, China). shNC was synthesized with the same bases of shRNA but with scrambled sequence. Lentiviral transfection and cell screening were performed according to manufacturer's instruction to establish the E2A stably downregulated and the stably NC transefected SW480 clones, i.e. SW480/shE2A and SW480/shNC. Transfection efficiency was evaluated by visual examination of the percentage of eGFP-expressing cells under fluorescence microscope (Olympus, Tokyo, Japan), western blot, and qRT-PCR.

### Transient transfection

Plasmids, pEZ-M29-E12 (plasmid encoding isoform E12), pEZ-M29-E47 (plasmid encoding isoform E47), pEZ-M29-NC (plasmid with negative control sequence) and vector controls were purchased from Genecopoeia (Rockville, MD, USA) and validated by sequencing; miRNA-320a mimics (double-strand RNA molecules mimicking miR-320a) and mimics NCs (negative control sequences based on C. elegans microRNAs have minimal sequence identity in human) were purchased from GenePharma (Shanghai, China). Cells of log phase were harvested and seeded in 6-well plates at a density of 4*10^5^ cells/well, 24 hours before transfection. Lipofectamine 2000 (Invitrogen, USA) was used for transfection, in accordance with manufacturer's instruction. Efficiency of transient transfection was examined by western blot or qRT-PCR. Cells transfected with vectors were used as control.

### Cell proliferation assay

Cell proliferation assay was performed with the Cell Counting Kit-8 (CCK8) (Dojindo, Kumamoto, Japan). Briefly, cells were digested with trypsin (Beyotime, Shanghai, China), washed with phosphate-buffered saline (PBS) twice, filtered with a strainer (BD Falcon), resuspended in RIPA 1640 medium, counted, and diluted to a final concentration of 5 cells/µl. Then cells were seeded in 96-well plates, 200 µl (1000 cells) per well, in sixplicate, and placed in the incubator for 6 days. Viable cells were quantified at each 24 h interval with CCK8, in accordance with manufacturer's protocol, and the absorbance at 450 nm was measured by using microplate reader (Epoch, BioTek).

### Cell cycle analysis

Before analysis, cells (2×10^6^) were harvested 48 hours after transient transfection, as well as the corresponding controls. Then, cells were washed twice with ice-cold PBS, fixed with 75% ethanol and stored at 4°C overnight. Upon analyzing, cells were washed with PBS twice and treated with RNase at 37°C for an hour, followed by staining with Propidium Iodide for 30 minutes in dark. Cell cycle analysis was then performed with flow cytometry (FACSCalibur, Becton Dickinson, MD, USA).

### Chromatin immunoprecipitation assay

EZ-ChIP™ Chromatin Immunoprecipitation Kit (Millipore, Billerica, MA, USA) was purchased to perform the ChIP assay, according to the manufacturer's protocol. Briefly, one 15 cm cell culture plate of SW480 cells (about 80% confluence) was fixed with 1% PFA (Sigma). Then cells were lysed, sonicated to shear DNA and immunoprecipitated with anti-E2A (Santa Cruz) and control antibody (IgG). After that, protein/DNA crosslink was reversed and DNA was purified for the following PCR, using Takara Ex Taq Hot Start Version (Takara).

### Statistical analysis

All statistical analyses were conducted by using the SPSS 15.0 software. The correlation of clinical factors with E2A expression was examined by Pearson correlation analysis. The effect of E2A on survival was estimated using the Kaplan-Meier curve and log-rank test. Univariate and multivariate Cox's proportional hazards model were used to evaluate the effects of E2A expression and clinicopathological parameters on 5-year OS and DFS, respectively. Student's t test was used to analyze differences between two groups and one-way ANOVA was employed in case of data consisted of more than two groups. A two-tailed value of *P*<0.05 was considered statistically significant.

## Results

### Expression of E2A correlated with CRC pathological stages

**Figure 1:**
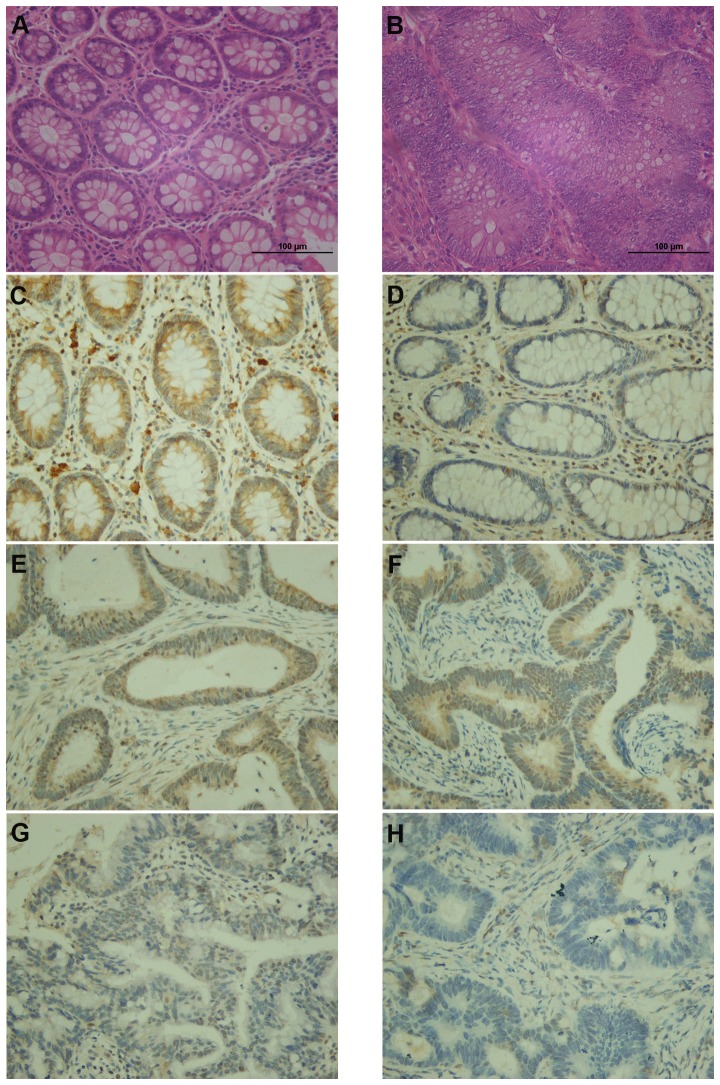
IHC staining of E2A in CRC tissues and normal mucosa. Representative images of IHC are shown as: (A) HE staining of normal mucosa; (B) HE staining of CRC tissue; (C) high E2A expression (brown color) presented as nuclear and cytoplasmic staining in normal mucosa; (D) low E2A expression in normal mucosa; (E–H) stage I (E) to stage II (F), III (G), and IV (H) CRC tissues with different E2A staining intensities; E2A appeared predominantly in nucleus. Images were taken under 200× magnification.

Firstly, we evaluated the expression of E2A protein in CRC tissues and normal mucosa by immunohistochemistry (Figure 1). Colorectal tumor specimens obtained from 98 CRC patients and normal mucosa available from 43/98 patients were examined. The demographic and clinicopathological parameters of all included patients were shown in Table 1. Of the 98 patients, high expression of E2A was detected in 35/43 normal mucosa and 39/98 tumor tissues (*P*<0.01). Staining of E2A in normal mucosa presented in nucleus and cytoplasm both, but in cancer tissues, E2A staining was predominantly nuclear (Figure 1). More importantly, expression of E2A decreased as CRCs advanced (Figure 1E–H). We then made a correlation analysis to study the association between E2A expression and the clinicopathological factors. As shown in Table 2, low expression of E2A was significantly associated with higher TNM stage (*P*<0.01) and larger tumor size (*P* = 0.035), but was not related to patients age (*P* = 0.761), gender (*P* = 0.655), tumor histology (*P* = 0.985), or tumor site (*P* = 0.120). Thus E2A was likely to be involved in the development and progression of CRC.

**Table 1:** Demographic and clinicopathological data.

Parameters	Case number
Age (years)	
40–59	44 (44.9%)
60–79	52 (53.1%)
≧80	2 (2%)
Gender	
Male	53 (54.1%)
Female	45 (45.9%)
Tumor histology	
Tubular adenocarcinoma	80 (81.6%)
Mucinous adenocarcinoma	17 (17.4%)
Papillary adenocarcinoma	1 (1%)
Tumor site	
Rectum and sigmoid	63 (64.3%)
Right colon	24 (24.5%)
Left colon	11 (11.2%)
Tumor size	
≦5 cm	50 (51.1%)
>5 cm	48 (48.9%)
TNM stage	
I	20 (20.4%)
II	33 (33.7%)
III	31 (31.6%)
IV	14 (14.3%)

**Table 2:** Association of E2A expression with demographic and clinicopathological parameters.

Parameters	Group	E2A expression		*P* value
		Low	High	
Age(years)	40–59	27	17	0.761
	60–79	30	22	
	≧80	2	0	
Gender	Male	33	20	0.655
	Female	26	19	
Tumor Histology	Tubular	48	32	0.985
	Mucinous	10	7	
	Papillary	1	0	
Tumor Site	Rectum and sigmoid	34	29	0.120
	Right colon	17	7	
	Left colon	8	3	
Tumor Size	≦5 cm	25	25	0.035
	>5 cm	34	14	
TNM stage	I	4	16	0.000
	II	19	14	
	III	23	8	
	IV	13	1	

### Low expression of E2A predicted poor prognosis of CRC patients

**Figure 2:**
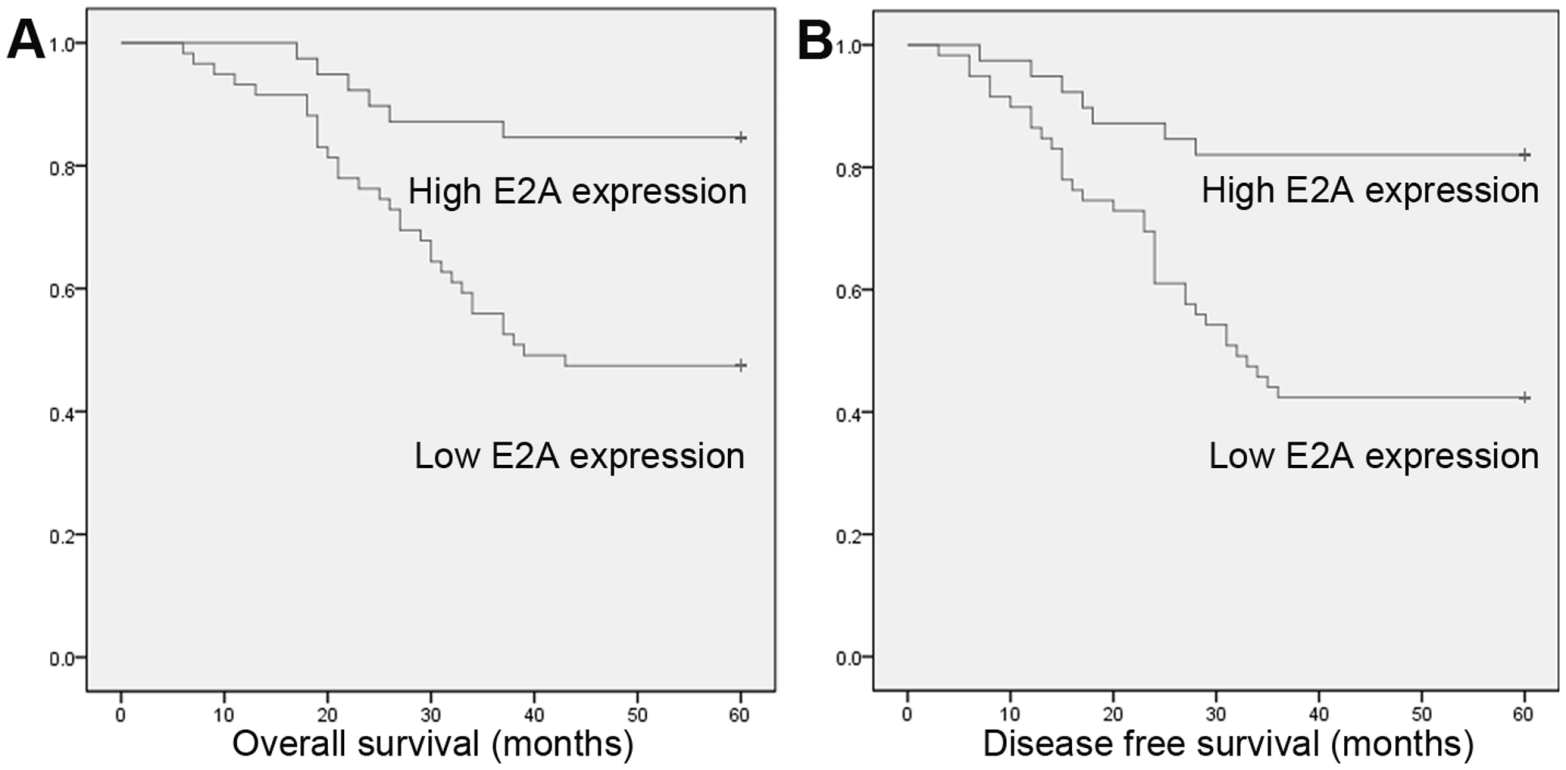
Kaplan-Meier survival curves for OS and DFS stratified by E2A expression. (A) OS for high and low E2A expression patients; (B) DFS for high and low E2A expression patients. Patients with high E2A expression have more favorable OS and DFS (*P* = 0.000, for OS and DFS both).

To investigate the prognostic value of E2A, we used Kaplan-Meier 5-year survival curve to show the differences in outcomes between low and high E2A expression CRC patients. As shown in Figure 2, patients with high E2A expression had longer 5-year overall survival (OS) and disease free survival (DFS) than patients with low expression. The 5-year OS and DFS for high E2A expression patients were 84.6% and 82.1%, and for patients with low E2A expression were 47.5% and 42.4% (*P* = 0.000, for OS and DFS both). Next, we employed the Cox regression model to examine the prognostic value of E2A. In univariate analysis, TNM stage and E2A expression were found to be predictive for OS and DFS; while in multivariate analysis, both factors also predicted worse clinical outcomes (Table 3). Hence E2A expression seemed to be a useful predictive factor for prognosis of CRC patients.

**Table 3:** Univariate and multivariate Cox regression analysis for OS and DFS in CRC patients.

Factors	Univariate			Multivariate		
	OR	95%CI	*P*	OR	95%CI	*P*
OS						
Age	0.999	0.968–1.030	0.934	0.991	0.961–1.022	0.578
Gender	1.045	0.547–1.995	0.895	1.177	0.606–2.286	0.631
Tumor Histology	1.142	0.724–1.803	0.567	1.193	0.746–1.909	0.462
Tumor Site	0.821	0.570–1.183	0.290	0.958	0.654–1.403	0.825
Tumor Size	1.270	0.665–2.424	0.469	1.071	0.553–2.076	0.839
TNM stage	2.547	1.737–3.732	0.000	2.270	1.512–3.407	0.000
E2A expression	4.275	1.781–10.261	0.001	2.592	1.022–6.570	0.045
DFS						
Age	0.994	0.965–1.024	0.688	0.985	0.956–1.015	0.325
Gender	1.085	0.587–2.005	0.795	1.216	0.649–2.276	0.541
Tumor Histology	1.094	0.717–1.670	0.677	1.119	0.723–1.731	0.614
Tumor Site	0.862	0.606–1.227	0.409	0.973	0.674–1.405	0.884
Tumor Size	1.381	0.745–2.559	0.306	1.203	0.634–2.282	0.571
TNM stage	2.409	1.684–3.445	0.000	2.189	1.494–3.207	0.000
E2A expression	4.065	1.799–9.186	0.001	2.534	1.061–6.049	0.036

### Inhibition of cell proliferation by E2A in CRC cells

**Figure 3:**
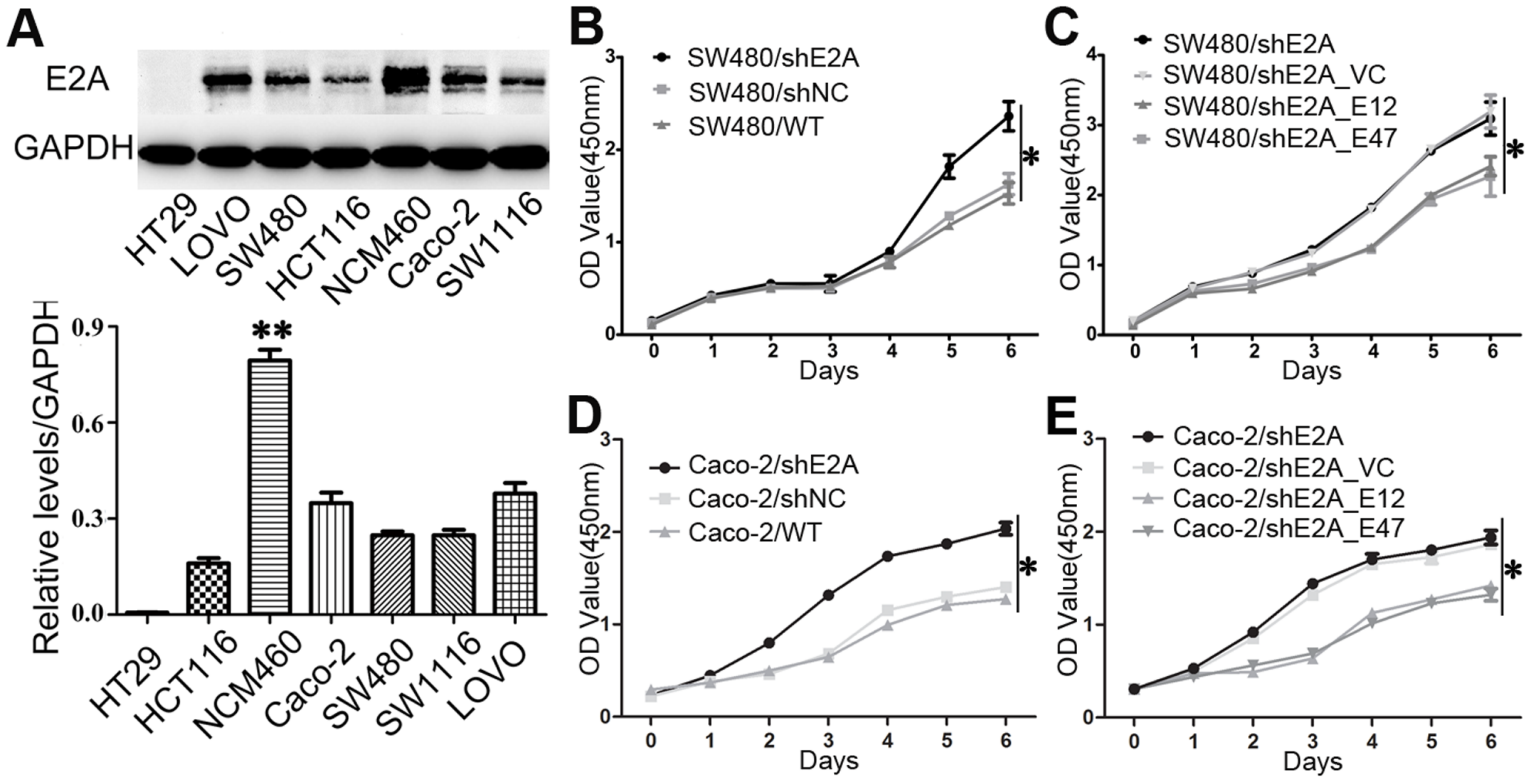
Knockdown of E2A promotes colon cancer cell growth. (A) Expression of E2A in normal human colon mucosal epithelium cell NCM460 and CRC cells lines was shown by western blot. GAPDH was used as loading control; (B) and (D) Cells with downregulated E2A expression (SW480/shE2A, Caco-2/shE2A) showed higher cell proliferation rate than that of the negative control (SW480/shNC, Caco-2/shNC) and wild type (SW480/WT, Caco-2/WT) cells; (C) and (E) Parental cells (SW480/shE2A, Caco-2/shE2A) were co-transfected with E12 or E47 plasmid (SW480/shE2A_E12 and SW480/shE2A_E47; Caco-2/shE2A_E12 and Caco-2/shE2A_E47) to restore E2A expression. Co-transfected cells showed decreased cell proliferation rate than that of the parental or vector control (SW480/shE2A_VC, Caco-2/shE2A_VC) transfected cells. All data represented as mean value ± SD from 3 independent experiments. (*, *P*<0.05).

Considering the clinical significance of E2A, we wanted to ask whether E2A played a role in the development of CRC. Six colon cancer cell lines, LOVO, HCT116, Caco-2, HT29, SW480, and SW1116 and normal human colon mucosal epithelium cell NCM460 were screened for E2A expression by using western blot (Figure 3A). Of the seven cell lines, NCM460 showed much higher E2A expression level than all other cancer cells. Taken together with its expression in CRC patients, E2A was significantly downregulated in CRC tissues and cells.

To directly investigate the function of E2A, we constructed E2A stably knocked-down clone, SW480/shE2A cells, and the negative control clone, SW480/shNC cells, with E2A/shRNA-LV and E2A/shNC-LV respectively. Knockdown efficiency was validated by western blot and qRT-PCR (Figure S1 A). Then, we detected the cell proliferation changes of SW480/shE2A and SW480/shNC cells with the MTT assay, using wild type SW480 cells (SW480/WT) as control. As shown in Figure 3B, SW480/shE2A showed higher proliferation rate than SW480/shNC and SW480/WT cells, indicating a suppressive role of E2A in proliferation. To further demonstrate the anti-proliferation role of E2A, we performed co-transfection in stable SW480/shE2A cells with two plasmids, pEZ-M29-E12 (encoding isoform E12) and pEZ-M29-E47 (encoding isoform E47) to offset the downregulation effect by shE2A. Co-transfection rescued the E2A expression of SW480/shE2A cells to normal level (Figure S1 A) and also restored the proliferation rate (Figure 3C). Moreover, transfection of E12 or E47 into wild type SW480 cells could significantly inhibit cell proliferation and the inhibition effects caused by E12 and E47 did not show any differences (Figure S1 B). To exclude the cell line dependent possibility, we constructed Caco-2/shE2A and Caco-2/shNC clones to repeat the above experiments and results showed E2A had the same anti-proliferation role in Caco-2 cells (Figure 3D & E). Additionally, we manipulated the E2A expression in NCM460 cells. E2A silencing and restoration also affected NCM460 cell growth in a suppressive manner (Figure S1 C). Conclusively, E2A might be a negative regulator of proliferation in colon cancer cells.

### E2A regulated cell cycle progression of SW480 cells

**Figure 4:**
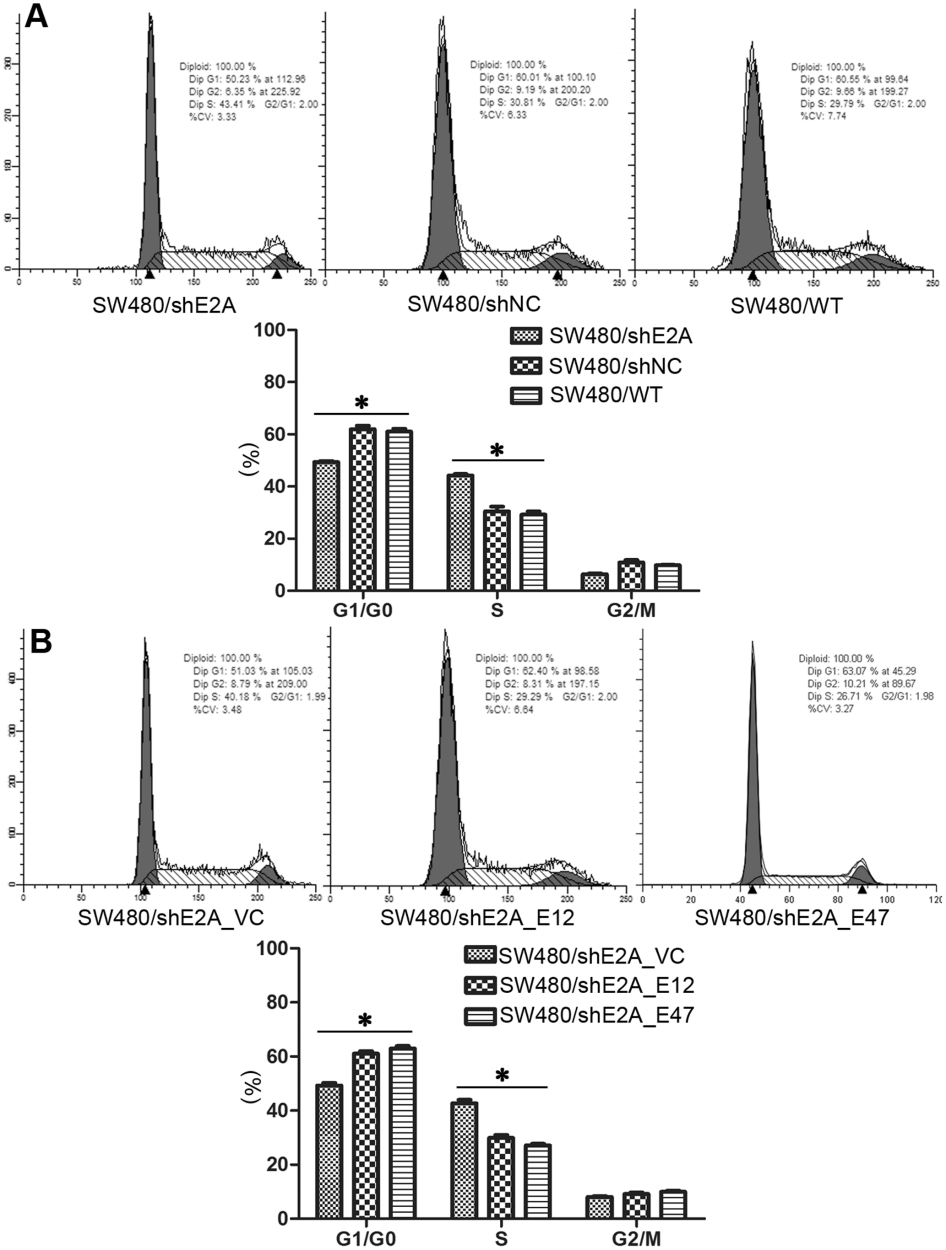
Effect of E2A on cell cycle progression. (A) Downregulation of E2A in SW480 cells led to an increase of cells at S phase and concomitantly a decrease of cells at G1/G0 phase; (B) Ectopic expression of E12 or E47 increased cells at G1/G0 phase of SW480/shE2A cells and reduced S phase cells. Data represents means ± SD from 3 independent experiments. (*, *P*<0.05).

Next we wanted to know the mechanisms through which E2A regulated SW480 cell proliferation. In previous publications, E2A was reported to be involved in cell cycle regulation [9], [23], [24]. Thus, we made cell cycle analysis by flow cytometry to detect potential changes after E2A downregulation and restoration. Consistently, the change of cell cycle explained alteration in cell proliferation: as shown in Figure 4A, cell cycle of SW480/shE2A cells differed from SW480/shNC and SW480/WT cells, with more cells progressed into S phase, indicating increased cell proliferation. When SW480/shE2A cells were co-transfected with either E12 or E47 plasmid, the cell cycle was normalized, which was in agreement with the restoration of cell proliferation (Figure 4B). Moreover, transfection of E12 or E47 into wild type SW480 resulted in a decrease of S phase cells (Figure S1 D). Similar regulation effects were also seen in NCM460 cells (Figure S1 E). Thus, E2A might regulate cell cycle to control cell proliferation.

### E2A controlled cell cycle by targeting miR-320a

**Figure 5:**
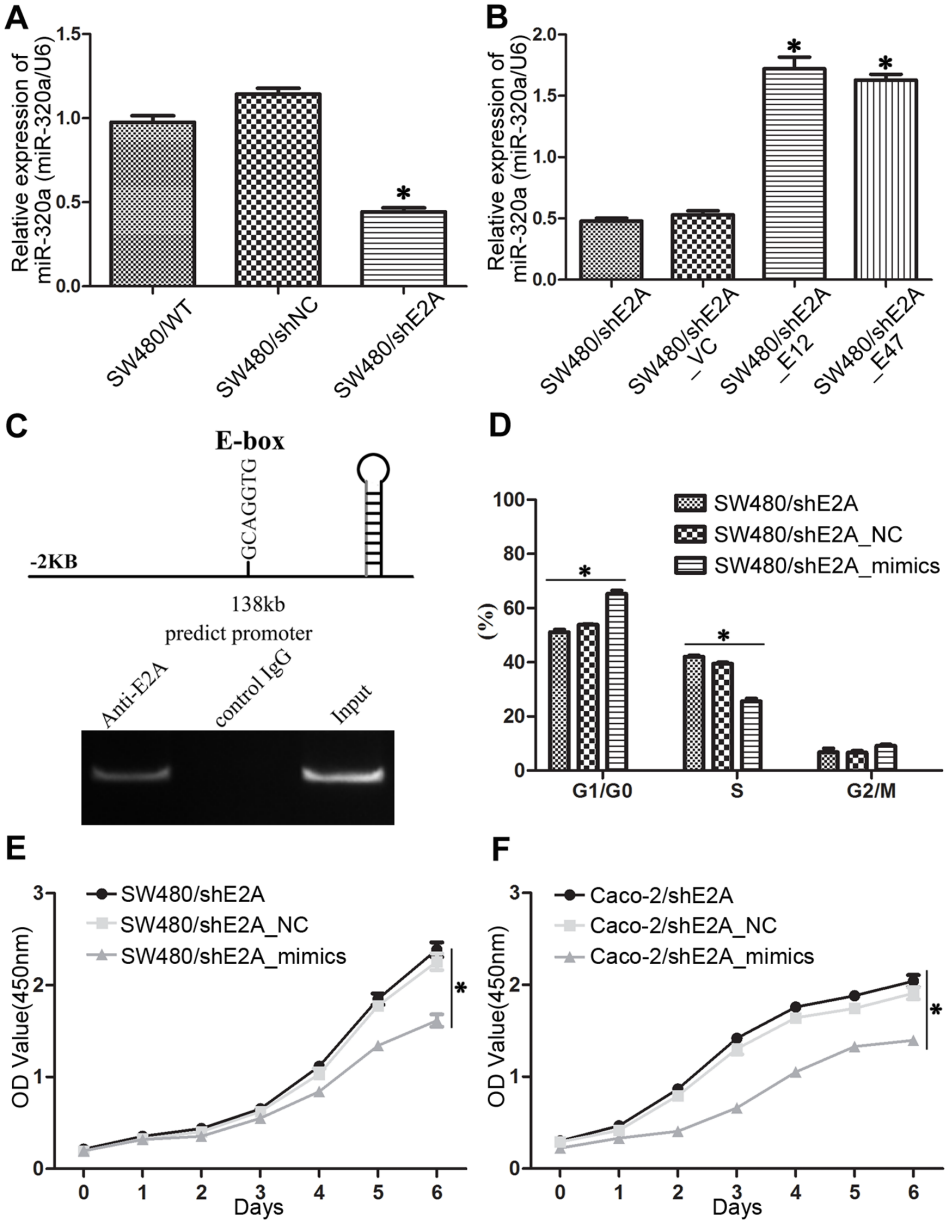
miR-320a is a direct target of E2A to regulate cell cycle and proliferation. (A) miR-320a expression was decreased in SW480/shE2A cells as shown by qRT-PCR; (B) Co-transfection of E12 or E47 into SW480/shE2A cells could normalize the expression of miR-320a; (C) Upper panel: sequence of the miR-320a gene showing the E-box, the predicted binding site of E2A; lower panel: ChIP assay showed E2A bound to miR-320a at its E-box, GCAGGTG; Co-transfection of miR-320a mimics reversed the cell cycle changes (D) and cell growth (E) of SW480/shE2A cells; (F) Co-transfection of miR-320a mimics decreased cell proliferation rate of Caco-2/shE2A cells. Data is expressed with the means ± SD from 3 independent experiments. (*, *P*<0.05).

The findings above further led us to investigate how E2A regulated cell cycle change. In recent decades, microRNAs (miRNAs) have been found to be involved in the pathogenesis of human diseases including cancer [25]. Unexpectedly, we found one miRNA, miR-320a, which was a metastasis suppressor [26], was regulated by E2A. In SW480/shE2A cells, the expression of miR-320a was downregulated (Figure 5A) and transfection of either E12 or E47 into this group of cells (Figure 5B) or into wild type SW480 cells (Figure S1 F) could upregulate miR-320a. To investigate whether miR-320a was a target directly regulated by E2A, we performed the chromatin immunoprecipitation (ChIP) assay. Results showed that E2A could bind to the E-box (GCAGGTG, at -138bp, upstream of miR-320a stemloop) of miR-320a, suggesting E2A may regulate the expression of miR-320a by targeting its promoter sequence directly (Figure 5C).

Then, we asked whether miR-320a was the target through which E2A regulated cell proliferation. By transfecting miR-320a mimics into SW480/shE2A cells, we found the cell cycle progression caused by E2A knockdown was arrested and cell proliferation rate was decreased (Figure 5D & E). Also, co-transfection of miR-320a mimics in Caco-2/shE2A cells inhibited cell proliferation rate (Figure 5F). Hence, E2A regulated cell proliferation by directly targeting miR-320a.

## Discussion

Despite great efforts having been made to improve the prevention and treatment of CRC, its morbidity and mortality still remain high: both were estimated to be the third among all cancers in USA, 2013 [27]. The tumorigenesis of CRC is a multiple process mediated by accumulating alterations in cell proliferation ability and a wide range of genetic disorders [28]. Based on these, recent studies have focused on finding new biomarkers and aberrant genetic features in CRC [29]. Here we showed E2A was a novel prognostic marker for CRC and miR-320a was a direct target through which E2A regulated colon cancer cell cycle to control cell proliferation.

The role of E2A in normal B and T cell development and leukemogenesis has been well studied [13], [30], [31] and it was also demonstrated to be involved in breast cancer, prostate cancer, gastric MALT and thymic lymphoma [15], [17], [18], [20]. Specially, E2A expression is associated with tumor cell differentiation and patient outcome in breast cancer [15] and tumor stages in prostate cancer [17]. Hence, E2A is likely to be a factor linked with tumor development and have prognostic value. In supporting of this, we found that E2A expression was significantly decreased in CRC tissues compared with normal mucosa and immunohistochemistry showed a gradually decreased scoring of positive E2A staining as clinical stages advanced, indicating a negative association of E2A with CRC development and a probably tumor-suppressive effect of E2A. In Cox regression analysis, we found low expression of E2A predicted poor prognosis of OS and DFS in CRC patients independent of age, gender, tumor site, tumor histology, and tumor size. These results, which suggested a suppressive role of E2A in CRC, were in consistent with the findings in lymphoma [18], [32] and leukemia [4], but contradicted with those found in breast and prostate cancer [15], [17]. Considering the multistep process of CRC carcinogenesis and different biological behaviors of tumors, the discrepancy could be understood at least partially.

Previous studies have shown the self-contradictive roles of E2A in proliferation: silencing of E2A in breast [15] and prostate cancer cells [17] inhibited cell growth, but in lymphoma, leukemia, hematopoietic stem cells, and CRC cancer cells, loss of E2A led to enhanced proliferation [7], [30], [32], [33]. Besides, enforced E47 overexpression inhibited cell growth of T cell ALL [34]. To clearly reveal the role of E2A in colon cancer cell proliferation, we constructed an E2A stably downregulated SW480 clone to investigate its effect. Consistent with prior results found in hematopoietic malignancy and CRC, we observed that downregulation of E2A significantly increased cell proliferation rate and co-transfection with either E12 or E47 plasmid could offset this effect, suggesting the direct role of E2A in regulating cell proliferation. Accordingly, cell cycle analysis showed a progression from G1/G0 phase to S phase in E2A downregulated cells. Although some studies showed cell cycle arrested at G1 phase in E2A deficient cancer cells [17], [23], our results were supportive to the pro-proliferation effect and in agreement with the findings of most studies which showed accelerated cell cycle progression after E2A deficiency [24], [35]–[39]. Taken together, knockdown of E2A promoted cell cycle progression and this resulted in increased cell proliferation. These findings provide partial, if not complete, insights into the tumor suppressive role of E2A in CRC.

As a transcriptional factor, E2A exerts its functions by regulating gene expression and studies have reported its downstream targets including p21, Id1, c-Myc, etc [17], [24], [36]. In our study, we found miR-320a was a target regulated by E2A in modulating cell cycle and cell proliferation. Human miR-320a is localized at chromosome 8p21.3, a locus of liver metastatic susceptibility [40], and it has been reported to be regulator of glycolysis [41] and dysregulated in cancer [42]. Studies have shown that miR-320a suppresses tumorigenesis and metastasis in CRC [26], [43], [44]. Unexpectedly, we identified the E-box of miR-320a as a binding site of E2A with TESS (Transcription Element Search System) and we indeed demonstrated that E2A bound to miR-320a using ChIP assay. Expression of miR-320a was regulated by E2A directly and more importantly, upregulation of miR-320a could reverse the changes caused by E2A knockdown, which further suggested it as a downstream effector of E2A. Yet, it remains unclear whether the effect of E2A on miR-320a is a global effect. Taken together, we propose that miR-320a is one of the targets through which E2A regulates cell cycle progression and cell proliferation.

In summary, we present convincing evidence showing that E2A is a prognostic factor for CRC patients and plays a tumor-suppressive role in CRC cells. Through binding directly to miR-320a, E2A regulates cancer cell cycle progression and controls cell growth. Yet, the role of E2A in solid tumors has not been fully understood and our findings only partially unveil the molecular targets and mechanisms of action of E2A. Hence, future studies are required to validate our findings and thoroughly elucidate the role of E2A in CRC.

## Supporting Information

Figure S1 (A) Left: E2A protein expression of wild type SW480, control SW480, and knocked-down SW480 cells. Right: Change of E2A expression in SW480 cells after transfection of shE2A, E12 or E47: shE2A reduced the expression of E2A in SW480, while E12 and E47 increased E2A expression in SW480/shE2A cells, relative to the controls; (B) Transfection of E12 or E47 inhibited SW480/WT cell growth; (C) E2A regulates cell growth in NCM460 cells; (D) Transfection of E12 or E47 increased G_0_/G_1_ phase of SW480/WT cells and decreased the S phase; (E) E2A regulates cell cycle progression in NCM460 cells; (F) Transfection of E12 or E47 upregulated the expression of miR-320a, compared to negative control. Data is expressed as the means ± SD from 3 separate experiments. (*, *P*<0.05; **, *P*<0.01).
(DOCX)
