# Supplementary material for: E2A Predicts Prognosis of Colorectal Cancer Patients and Regulates Cancer Cell Growth by Targeting miR-320a
Source: PLoS One. 2014 Jan 13;9(1):e85201. doi: 10.1371/journal.pone.0085201 (PMC3890311; doi:10.1371/journal.pone.0085201)

Figure S1 (A) Left: E2A protein expression of wild type SW480, control SW480, and knocked-down SW480 cells. Right: Change of E2A expression in SW480 cells after transfection of shE2A, E12 or E47: shE2A reduced the expression of E2A in SW480, while E12 and E47 increased E2A expression in SW480/shE2A cells, relative to the controls; (B) Transfection of E12 or E47 inhibited SW480/WT cell growth; (C) E2A regulates cell growth in NCM460 cells; (D) Transfection of E12 or E47 increased G_0_/G_1_ phase of SW480/WT cells and decreased the S phase; (E) E2A regulates cell cycle progression in NCM460 cells; (F) Transfection of E12 or E47 upregulated the expression of miR-320a, compared to negative control. Data is expressed as the means ± SD from 3 separate experiments. (*, *P* <0.05; **, *P* <0.01)


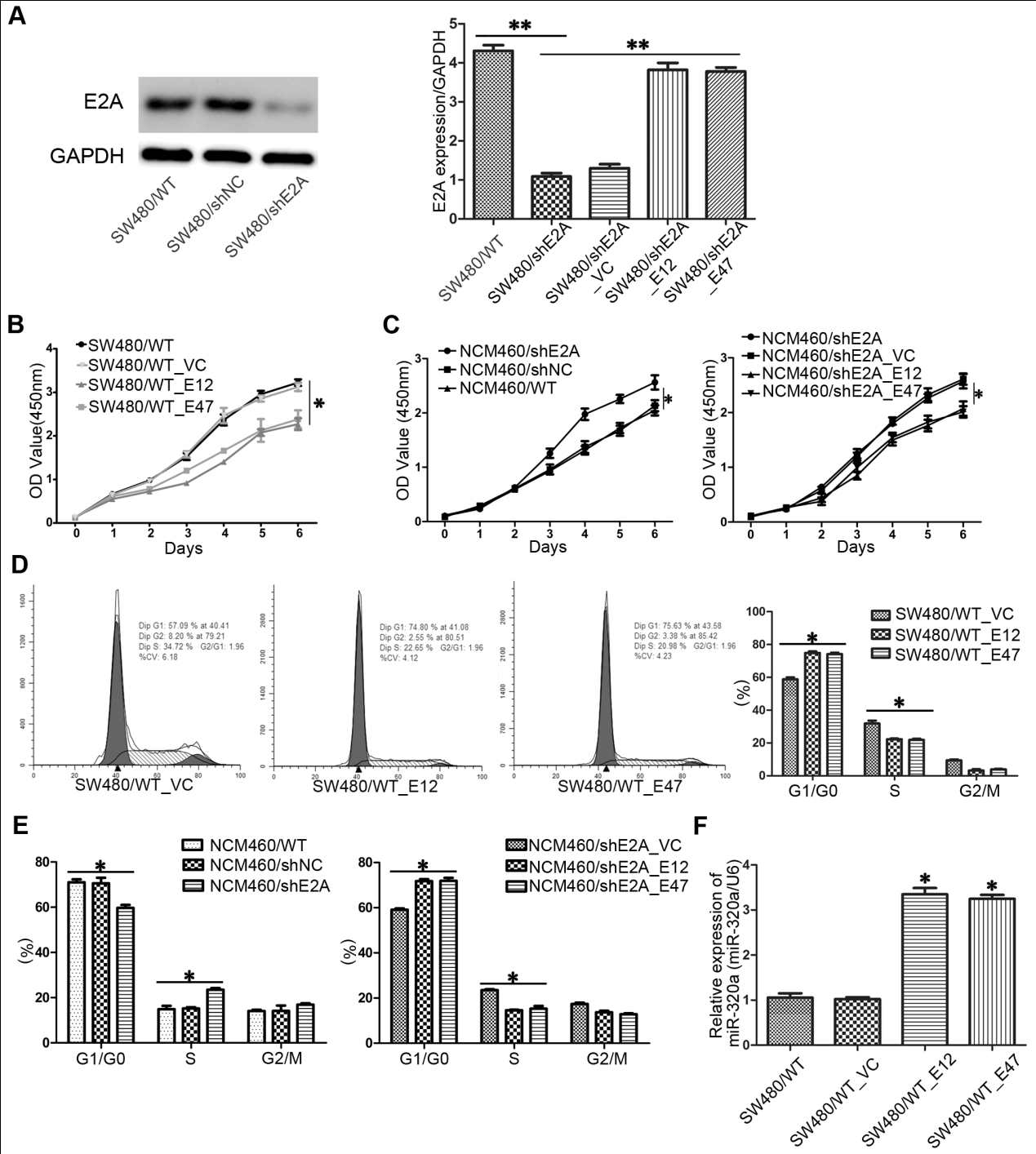

Supplement: Figure S1 — (A) Left: E2A protein expression of wild type SW480, control SW480, and knocked-down SW480 cells. Right: Change of E2A expression in SW480 cells after transfection of shE2A, E12 or E47: shE2A reduced the expression of E2A in SW480, while E12 and E47 increased E2A expression in SW480/shE2A cells, relative to the controls; (B) Transfection of E12 or E47 inhibited SW480/WT cell growth; (C) E2A regulates cell growth in NCM460 cells; (D) Transfection of E12 or E47 increased G0/G1 phase of SW480/WT cells and decreased the S phase; (E) E2A regulates cell cycle progression in NCM460 cells; (F) Transfection of E12 or E47 upregulated the expression of miR-320a, compared to negative control. Data is expressed as the means ± SD from 3 separate experiments. (*, P<0.05; **, P<0.01). (DOCX) [file pone.0085201.s001.docx]
